# Supplementary material for: The long non‐coding RNA Paupar promotes KAP1‐dependent chromatin changes and regulates olfactory bulb neurogenesis
Source: EMBO J. 2018 Apr 16;37(10):e98219. doi: 10.15252/embj.201798219 (PMC5978383; doi:10.15252/embj.201798219)
Supplement: Supplementary file 1 — Expanded View Figures PDF [file EMBJ-37-e98219-s001.pdf]

Expanded View Figures

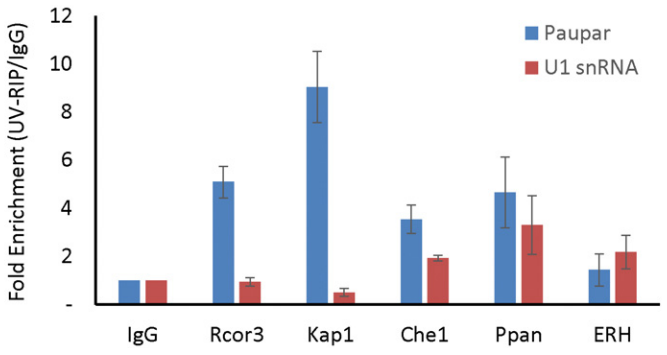

Figure EV1. Characterisation of *Paupar* lncRNA–protein interactions using UV-RNA-IP.

Nuclear extracts were prepared from UV cross-linked N2A cells and immunoprecipitated using either the indicated antibodies or a rabbit IgG control antibody. Associated RNAs were stringently washed and purified. The levels of *Paupar* and *U1snRNA* were detected in each UV-RIP using qRT–PCR. Results are presented as fold enrichment relative to control antibody. Mean values ± SEM, $N = 3$.

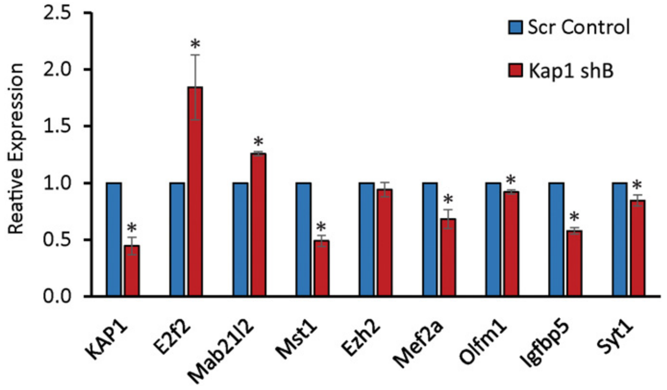

Figure EV2. Validation of the specificity of the KAP1 regulated gene set.

N2A cells were transfected with an additional Kap1 targeting shRNA expression vector shB-Kap1 or a scrambled control plasmid. Three days later, cells were harvested and expression of the indicated KAP1 targets analysed using RT–qPCR. Samples were normalised using *Gapdh*, and the results are presented relative to the control. Results are presented as mean values ± SEM, $N = 3$; $^*P < 0.05$, one-tailed *t*-test, unequal variance.

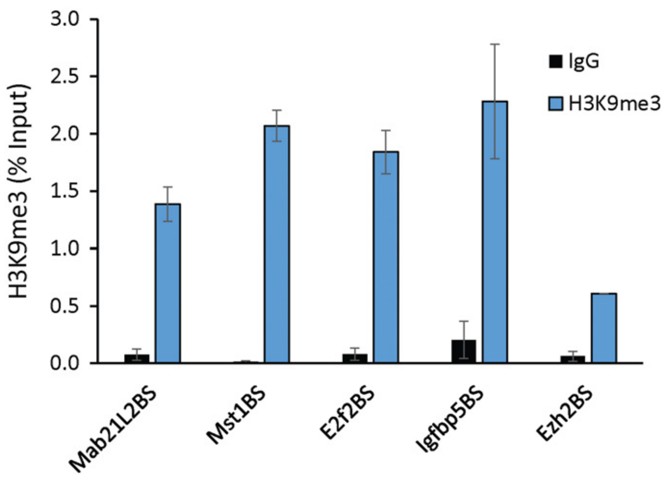

Figure EV3. *Paupar*-KAP1–PAX6 bound sequences within the regulatory regions of the *Mab21L2*, *Mst1*, *E2f2*, *Igfbp5* and *Ezh2* genes are enriched in H3K9me3 modified chromatin.

ChIP assays were performed in N2A cells using either histone H3K9me3 or anti-rabbit IgG control antibody. DNA fragments were amplified using qPCR. % input was calculated as $100 \times 2^{(C_t\text{Input}-C_t\text{IP})}$. Results are presented as mean values ± SEM, $N = 4$.

© 2018 The Authors

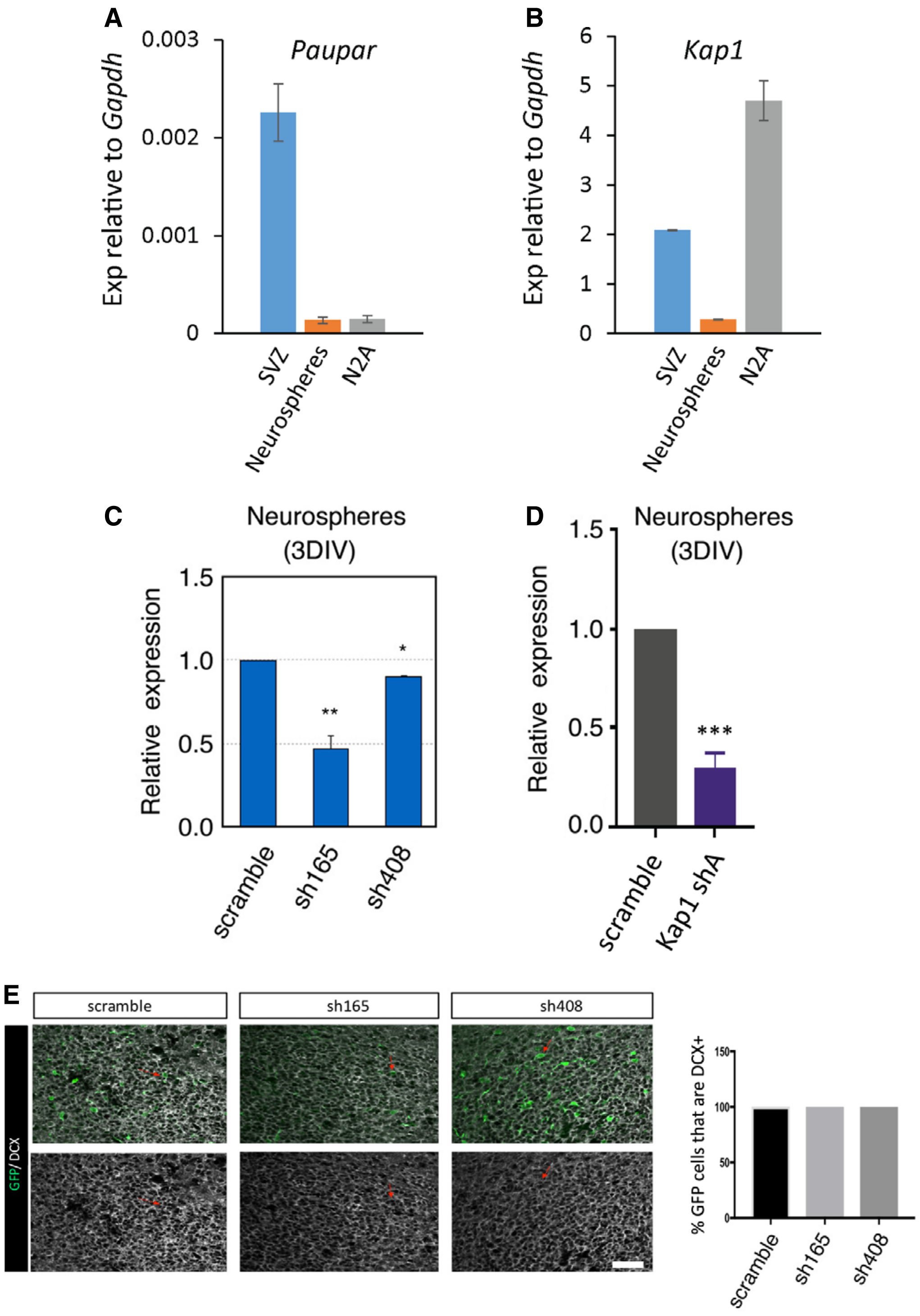

Figure EV4. *Paupar* and *Kap1* SVZ analysis.

A, B *Paupar* and *Kap1* transcript levels were determined using RT–qPCR in the P4 SVZ and in tertiary neurospheres prepared from the P4 SVZ.

C, D Neurosphere cultures were transfected with the indicated *Paupar* or *Kap1* targeting shRNA expression vectors or a non-targeting control. *Paupar* and *Kap1* expression was quantified using qRT–PCR 3 days later and normalised using *Gapdh*. The results are presented relative to the scrambled control (set at 1).

E GFP and DCX co-labelling in the OB 7 dpe. Small red arrows show examples of co-labelled cells. Data information: Data are shown as mean ± SEM. $N = 3$. In (E), there are no error bars because 100% of all GFP^+ cells in the OB were DCX^+. Scale bar represents 30 μm. The same scale is used for all images. *$P < 0.05$, **$P < 0.01$, ***$P < 0.001$, one-tailed *t*-test, unequal variance.

© 2018 The Authors

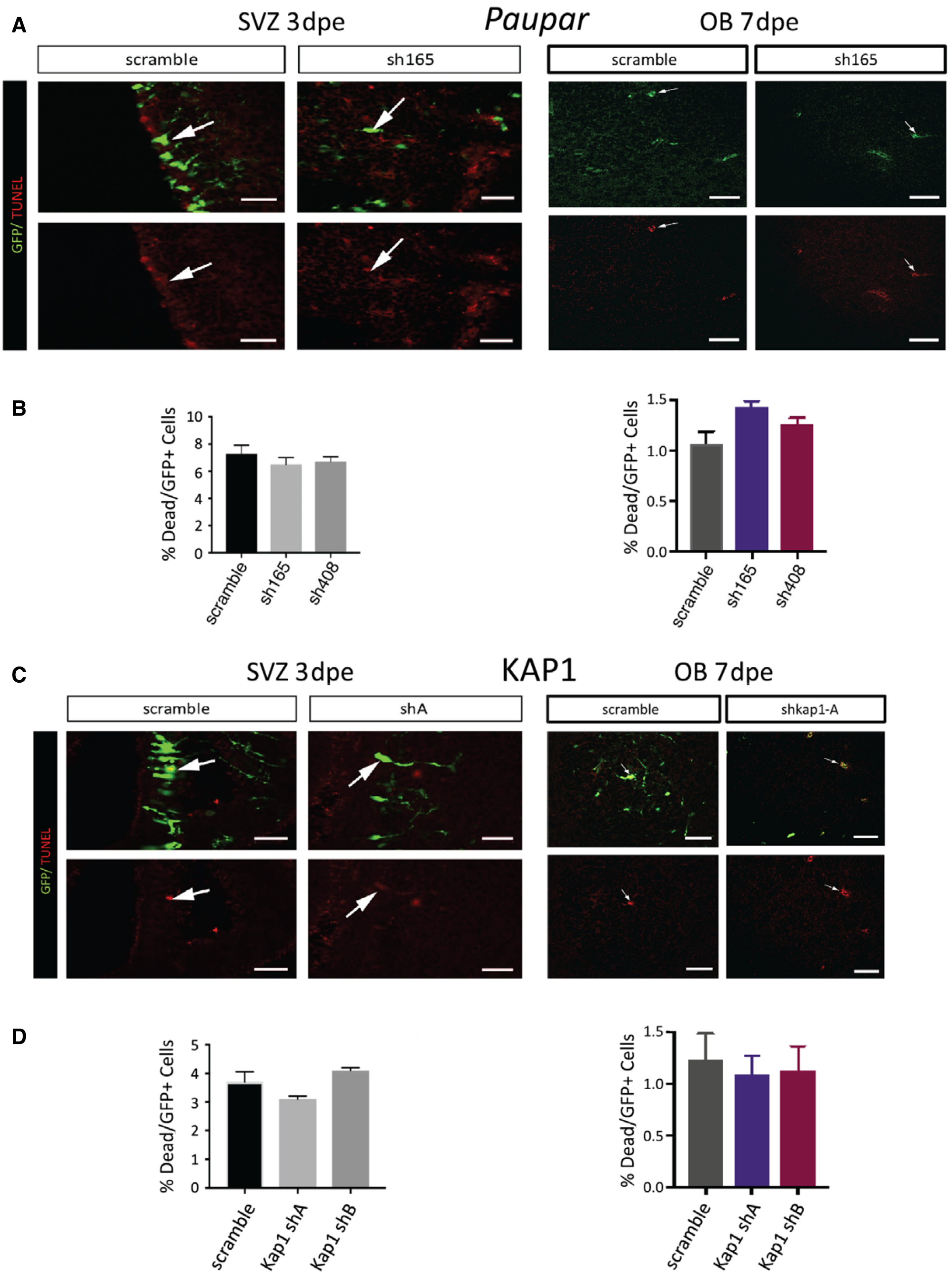

Figure EV5. ***Paupar*- and *Kap1*-mediated decrease in neuronal numbers is not due to increased cell death.**

A–D Quantification of TUNEL$^+$ cells after *Paupar* (A, B) or KAP1 (C, D) depletion to detect apoptosis in the SVZ at 3 dpe and OB at 7 dpe. Arrows show examples of co-labelled TUNEL$^+$ and GFP$^+$ cells. N = 3. Data are presented as mean ± SEM and analysed by one-way ANOVA. Scale bars represent 30 μm.

© 2018 The Authors
