# Supplementary figures and images for: The long non‐coding RNA Paupar promotes KAP1‐dependent chromatin changes and regulates olfactory bulb neurogenesis
Source: EMBO J. 2018 Apr 16;37(10):e98219. doi: 10.15252/embj.201798219 (PMC5978383; doi:10.15252/embj.201798219)

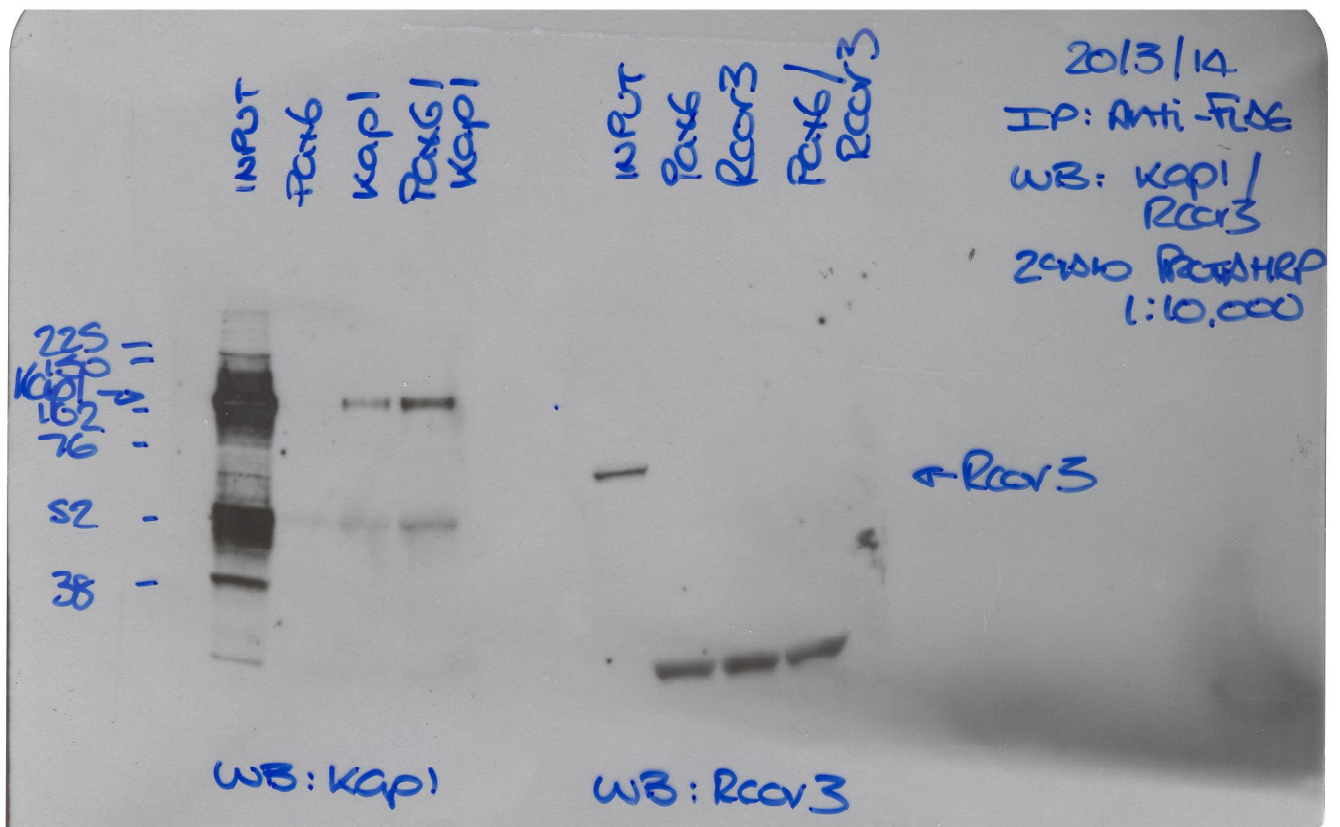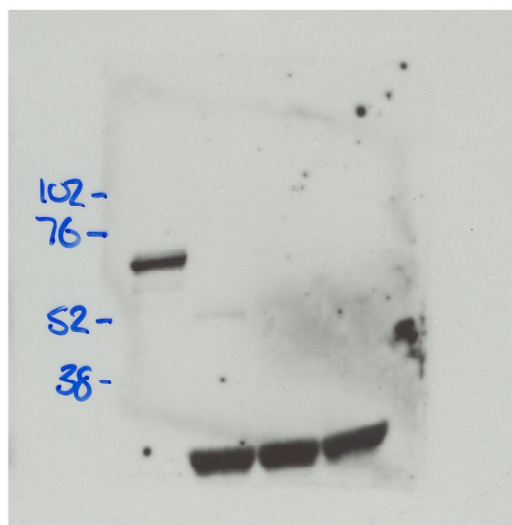

Reprobed  
 PAX6

150 225  
 102 102  
 52 52  
 38 38  
 38

← from exp 2013

Supplement: Supplementary file 8 — Source Data for Figure 1 [file EMBJ-37-e98219-s007.pdf]

Figure 3E

KAP1

ACTIN

Scrambled

sh408

sh165

sh1849

Scrambled

sh408

sh165

sh1849

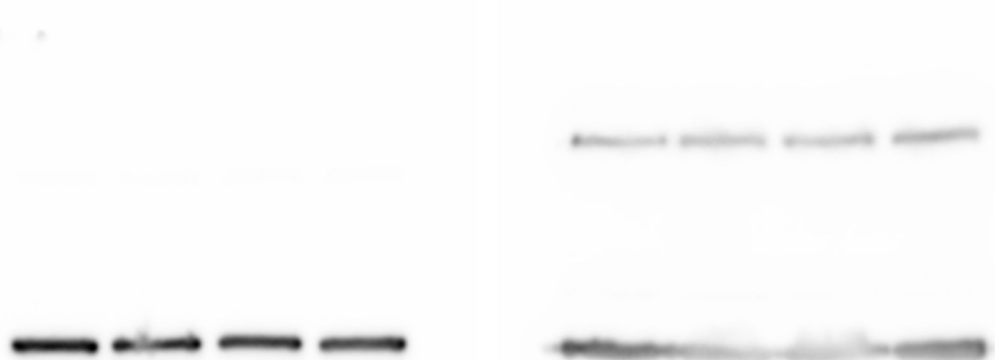

Figure 3F

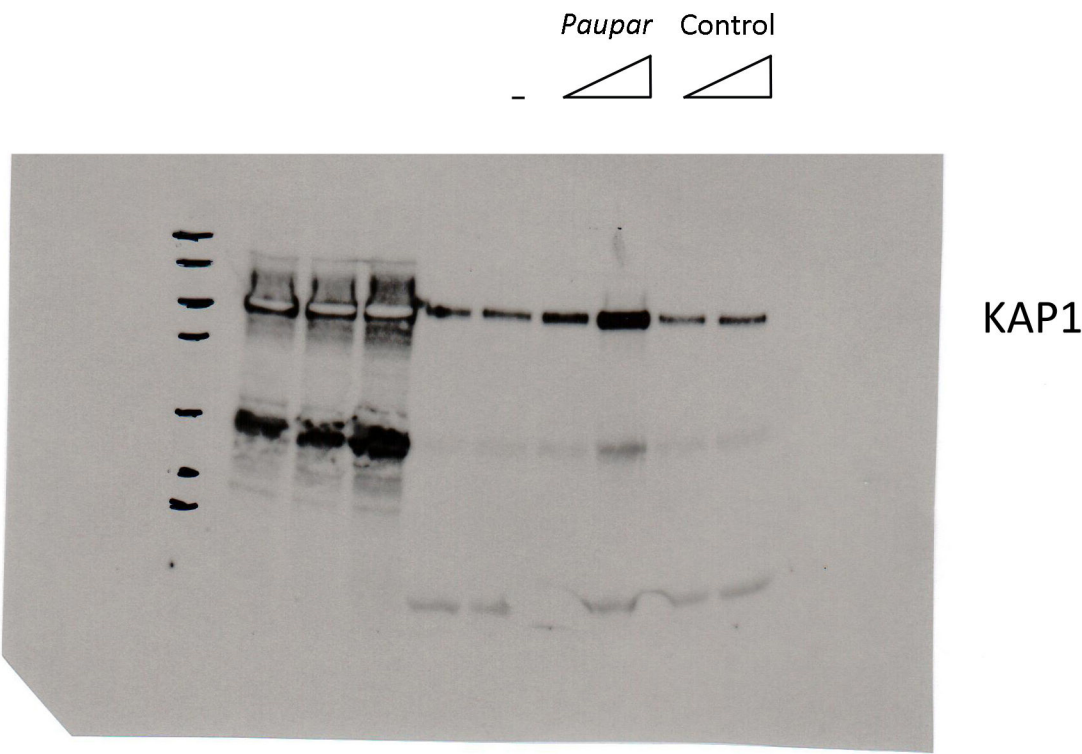

Figure 3F

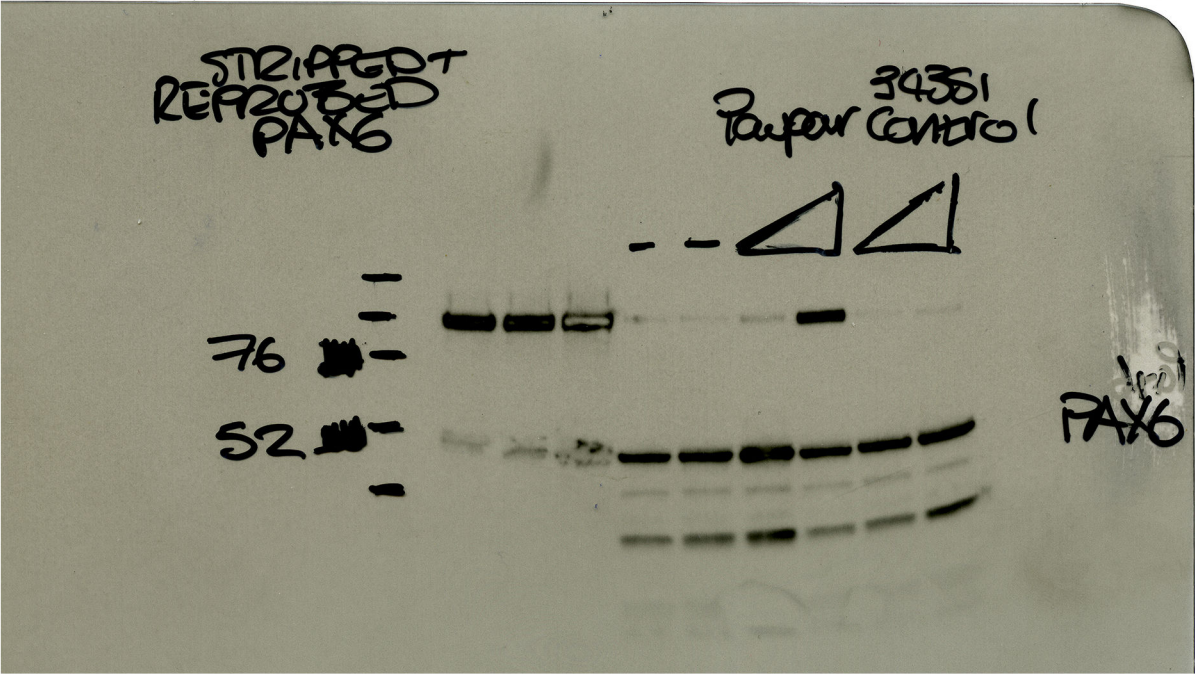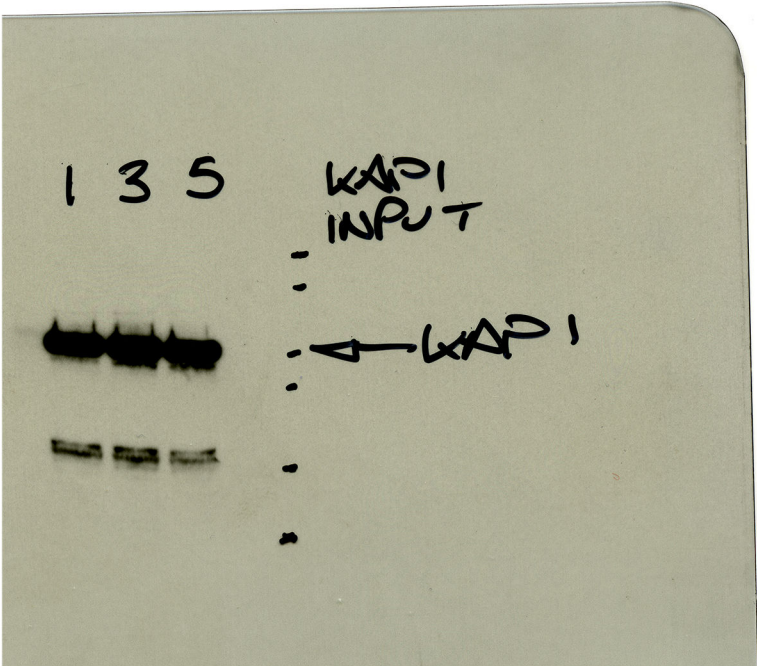

Supplement: Supplementary file 9 — Source Data for Figure 3 [file EMBJ-37-e98219-s008.pdf]
